# Differentiating midazolam over-sedation from neurological damage in the intensive care unit

**DOI:** 10.1186/cc3010

**Published:** 2004-12-14

**Authors:** Catherine A McKenzie, William McKinnon, Declan P Naughton, David Treacher, Graham Davies, Gary J Phillips, Philip J Hilton

**Affiliations:** 1 Senior Pharmacist, Intensive Care Medicine, Department of Pharmacy, Guy's and St. Thomas' Hospital, London, UK; 2 Senior Scientist, Renal Laboratory, St. Thomas' Hospital, London, UK; 3 Senior Lecturer, School of Pharmacy and Biomolecular Sciences, University of Brighton, Brighton, UK; 4 Consultant Intensivist, Intensive Care Unit, Guy's and St. Thomas' NHS Trust, London, UK; 5 Academic Director of Clinical Studies, School of Pharmacy and Biomolecular Sciences, University of Brighton, Brighton, UK; 6 Research Fellow, School of Pharmacy and Biomolecular Sciences, University of Brighton, Brighton, UK; 7 Director of the Renal Laboratory, Intensive Care Unit, Guy's and St. Thomas' NHS Trust, London, UK

**Keywords:** 1-hydroxmidazolam glucuronide, midazolam, neurological coma

## Abstract

**Introduction:**

Midazolam is used routinely to sedate patients in the intensive care unit (ICU). We suspected that midazolam over-sedation was occurring in the ICU of the Guy's and St. Thomas' Trust and that it could be difficult to differentiate this from underlying neurological damage. A sensitive assay for detecting midazolam and 1-hydroxymidazolam glucuronide (1-OHMG) in serum was developed and applied in the clinical setting.

**Methods:**

In the present study we evaluated a series of cases managed in a mixed medical, surgical and trauma ICU. Serum was collected from 26 patients who received midazolam, were 'slow to wake' and in whom there was suspicion of neurological damage. Patient outcome was followed in terms of mortality, neurological recovery and neurological damage on discharge.

**Results:**

Out of 26 patients, 13 had detectable serum levels of midazolam and/or 1-OHMG after a median of 67 hours (range 36–146 hours) from midazolam cessation. Of these 13 patients in whom midazolam/1-OHMG was detectable, 10 made a full neurological recovery. Of the remaining 13 patients with no detectable midazolam/1-OHMG, three made a full neurological recovery; 10 patients were subsequently found to have suffered neurological damage (*P *< 0.002), eight of whom died and two were discharged from the ICU with profound neurological damage.

**Conclusion:**

These findings confirm that prolonged sedation after midazolam therapy should be considered in the differential diagnosis of neurological damage in the ICU. This can be reliably detected by the assay method described. The effects of midazolam/1-OHMG persist days after administration of midazolam has ceased. After prolonged sedation has been excluded in this patient group, it is highly likely that neurological damage has occurred.

## Introduction

Midazolam is an intravenous sedative that is commonly used during ventilation in critical illness. It is often regarded as the sedative of choice in the intensive care unit (ICU). According to the findings of our recent electronic survey (93% respondents) [1], midazolam is still routinely used in the UK as a sedative in ICUs.

When used as a single dose, midazolam's pharmacological characteristics appear favourable, with a rapid onset of action and a short plasma elimination half-life. Midazolam is 94–98% bound to plasma albumin and has a volume of distribution of 1.7 l/kg in healthy individuals [2]. It is extensively metabolized first via cytochromes p450, 3A4 and 2B6 to 1-hydroxymidazolam, before undergoing glucuronidation to form 1-hydroxymidazolam glucuronide (1-OHMG), which has sedative properties and is excreted in the urine [3,4]. A wide interpatient variability in the pharmacokinetic properties of midazolam in critically ill patients with multiple organ failure has been reported [5], which can lead to prolonged sedation after midazolam therapy is stopped. However, there are other important causes of patients being 'slow to wake'; of these, it is most important to identify severe neurological damage. Patients with multiple organ failure are at high risk for neurological damage because they frequently have episodes of hypotension and dysrhythmia, and may have significant coagulopathy during the course of their critical illness.

We suspected that some patients in our ICU, particularly those with renal impairment, were becoming over-sedated with midazolam and the active metabolite 1-OHMG, and that this was complicating the neurological assessment of 'slow to wake' patients. We previously developed a rapid assay for measuring midazolam and its glucuronide metabolite simultaneously [1]. This short report describes the usefulness of this assay for identifying midazolam over-sedation and its potential use as a predictor of eventual neurological recovery.

## Methods

The assay was available for clinical application in the ICU. To differentiate between midazolam over-sedation and neurological damage, consultant intensivists requested detection of midazolam and 1-OHMG in serum. This request was normally made during the morning ICU ward round. The patients studied were those who had received intravenous midazolam therapy by continuous infusion either before (e.g. in operating theatres) or during the course of their ICU admission, and who were 'slow to wake' and in whom there was clinical suspicion of neurological damage.

Arterial blood (2 ml) was collected from each patient via an *in situ *arterial catheter. The time of sample collection and the midazolam administration history, including cessation time, were recorded. A specific assay utilizing high-performance liquid chromatography coupled to mass spectrometric detection was used for simultaneous detection and quantification of midazolam and 1-OHMG [1]. Mass spectrometry allowed identification of midazolam and 1-OHMG individually based on their isotopic patterns. The studies were performed on the basis of clinical need, and in all cases they were requested by the consultant intensivist, normally during the morning ward round. The quantified serum level of midazolam and 1-OHMG could be reported to the medical team after a minimum of 2 hours so that they could consider the findings in their decisions regarding further clinical intervention. In practice, morning requests were available for interpretation by the evening round.

### Unit characteristics

The ICU at Guy's and St. Thomas' National Health Service Trust is a 30-bed, level 3 unit that serves a mixture of medical, surgical, trauma, oncology and haematology patients. It has an average of 100 admissions per calendar month. For the year from March 2003 to April 2004, the mean Acute Physiology and Chronic Health Evaluation II score (day 1) was 18.5 ± 7.3, with a hospital mortality of 32.5% and a median length of stay of 5 days (variance 189.5, maximum 246).

### Patient characteristics

All patients appeared to be deeply sedated at the time that the sample was taken, with a Glasgow Coma Scale score of less than 5. They were considered 'slow to wake' from either a pharmacological and neurological cause if, in the absence of a focal neurological deficit, consciousness did not return within 36 hours of stopping sedation. Patients were deemed to have regained consciousness if they both opened their eyes and moved their limbs in response to commands.

Studies were conducted in 26 patients who had received midazolam sedation therapy by continuous intravenous infusion and in whom neurological damage was considered clinically possible (e.g. a hypoxic event was noted during cardiac surgery). The mean age of these patients was 63 ± 16 years, and the median time from cessation of midazolam therapy to serum collection was 67 hours (range 36–146 hours). The median daily midazolam dose was 4 mg/hour (range 2–20 mg/hour). The reasons for ICU admission are described in Table 1.

We followed the clinical outcomes of these patients in terms of mortality, neurological recovery and neurological damage on discharge. If no midazolam or 1-OHMG was detected, then a series of standard clinical and diagnostic tests was undertaken to determine whether neurological damage was likely. These included the response to painful stimuli and computed tomography of the head. In patients in whom midazolam or 1-OHMG was detected, tests were deferred until either the patients awoke or levels became undetectable.

## Results

Midazolam and/or 1-OHMG were detected in the serum of 13 of the 26 patients (referred to as the midazolam-positive group). Of these 13 patients, 10 made a full neurological recovery; nine of these patients were discharged from the ICU and one later died as a result of critical illness but with intact neurological function. The remaining three patients died without regaining consciousness as a result of neurological damage.

In contrast, neurological damage was observed in 10 of the remaining 13 patients who had no detectable serum concentrations of midazolam and/or 1-OHMG (midazolam-negative group). Midazolam-positive patients were significantly less likely to have experienced neurological damage (χ^2 ^test [degrees of freedom = 1]: *P *< 0.002).

Twelve of the midazolam-positive patients had serum midazolam concentrations between 16 and 650 ng/ml, with a median value of 30 ng/ml, whereas the remaining patient's level exceeded the upper limit of the assay (3000 ng/ml). 1-OHMG was detected at a mean of 6800 ± 3432 ng/ml (range 3121–11,525 ng/ml) in the serum of six of the 13 midazolam-positive patients. All six of these patients exhibited a degree of renal impairment (defined as serum creatinine >130 μmol/l; Table 1), four of whom required renal replacement therapy in the form of continuous venovenous haemofiltration (employing an ultrafiltration rate of between 1500 and 3000 ml/hour). 1-OHMG was not detected in any of the midazolam-negative patients.

Of the 13 midazolam-negative patients, eight died without regaining consciousness as a result of neurological damage, and two were discharged from the ICU with significant neurological impairment and required prolonged neurological rehabilitation. None of these 10 patients had responded appropriately to painful stimuli when in the ICU. In addition, in seven of these patients structural neurological damage was detected by computed tomography scan. Only three out of 13 patients in this group of midazolam-negative patients left the ICU with no neurological deficit.

### Other sedative and opiate agents

Out of 26 patients, 15 were administered fentanyl by continuous intravenous infusion at a dosage between 0 and 300 μg/hour. In the 15 patients the fentanyl infusion was ceased at a minimum of 56 hours and a maximum of 120 hours before sample collection. In 25 of the 26 patients we could find no documented evidence of administration of sedative and opiate agents for a minimum of 36 hours before serum sample collection. The remaining patient, in the midazolam-negative group, was receiving 30 mg/day of the sedating antihistamine chlorphenamine; this was one of the three patients who were discharged from the ICU with neurological function intact.

## Discussion

In this study, midazolam with or without 1-OHMG was detected in half of the 'slow to wake' patients, in whom testing was requested after a mean time from therapy cessation of 3 days. In one patient, in whom there was no record of midazolam administration in the ICU, a level of 200 ng/ml was recorded. It later transpired that a large dose of midazolam had been administered in the operating theatre more than 96 hours earlier. Detection of 1-OHMG in renal impairment confirmed that 1-OHMG accumulates in the presence of renal failure. Furthermore, its presence in high serum concentrations (3121–11,525 ng/ml) in the face of midazolam levels below the therapeutic range, normally quoted in the critically ill of 100–1000 ng/ml [5], while the patient remained deeply sedated concurs with earlier reports [3,4] that 1-OHMG has a sedative effect and contributes to prolonged sedation in renal impairment. Other investigators have reported the presence of 1-OHMG in the absence of midazolam [3,4], but we did not observe this and suspect that it was because the assay we used is able to detect very low concentrations of midazolam.

Our findings suggest that serum levels of midazolam and/or 1-OHMG in 'slow to wake' patients may be used to aid differentiation between prolonged sedation and neurological damage. Patients found to be midazolam positive using this rapid assay were significantly less likely to have suffered neurological damage. Correct discrimination between neurological damage and prolonged sedation was made for 20 out of 26 patients, indicating a high degree of accuracy. Clearly, the possibility that midazolam-positive patients also have neurological damage remains and must be excluded if these patients do not awaken when serum concentrations of benzodiadepines have fallen to undetectable levels. Additionally, in the midazolam-negative group three patients were discharged with neurological function intact. This of course does not exclude a neurological cause of the coma that had fully resolved on discharge. One patient was receiving the sedating antihistamine chlorphenamine (30 mg/day intravenously) and did not regain full consciousness until it was stopped. In the remaining two patients no other clinical cause of the coma was apparent.

The only other agent used routinely in these patients that could have significantly contributed to their reduced level of consciousness was the intravenous opiate fentanyl. Although fentanyl is known to accumulate in critical illness [6], we could find no evidence of accumulation for longer than 36 hours [7], and, because our group of patients had not received the drug for more than 2 days before sampling, it was not thought to contribute to the patients being 'slow to wake'.

Arguably, the most important finding is that over three-quarters of the 'slow to wake' patients with no detectable serum midazolam/1-OHMG either died or were discharged from the ICU with profound neurological damage, whereas more than three-quarters of those with detectable midazolam/1-OHMG went on to make a full recovery. This observation suggests that prolonged sedation occurs after midazolam therapy and that it can be difficult to differentiate this from neurological damage in the acutely ill patient. The exclusion of midazolam or its metabolite 1-OHMG should be confirmed either by assay detection, as we describe, or by using the short-acting benzodiazepine antagonist flumazenil before a formal diagnosis of neurological damage is made. There are reports [3,8] in the literature of successful reversal of benzodiazepine sedation in critical illness using flumazenil, but we rarely use it in our unit because we find it to be nonspecific, short acting and able to induce seizures [9].

We recommend that use of alternatives to midazolam be considered in this patient group whenever possible, and that if its use is considered essential then steps should be taken to exclude the continuing presence of the drug or its metabolite before an opinion regarding neurological damage is formed.

These findings have led to a change in prescribing practice in our ICU. We no longer use midazolam for sedation, and our sedation policy is now based on administering propofol or lorazepam. This view is also supported by the Society of Critical Care Medicine's most recently published guidelines [10], which recommend use of lorazepam for sedating most patients via intermittent or continuous infusion and use of propofol for short-term sedation, and that midazolam be reserved for rapid control of agitated patients and for short-term sedation. As a consequence, we were unable to conduct a more formal study of midazolam's role in over-sedation or extend the study to a larger group of patients.

## Conclusion

The results of this investigation confirm that prolonged sedation from midazolam or 1-OHMG should always be considered in the differential diagnosis of neurological damage in critically ill patients who have received midazolam. This can be accurately detected using the assay method described. The sedative effects of midazolam/1-OHMG can persist for days after stopping administration of midazolam. If prolonged sedation can be excluded in these patients, then it is highly likely that neurological damage has occurred.

## Key messages

• 	In some patients midazolam is metabolized to its glucuronide, which has sedative properties.

• 	Prolonged sedation resulting from this metabolite should be considered when making a differential diagnosis of neurological damage in 'slow to wake' patients.

• 	Measurement of midzolam and its metabolite in slow to wake patients will aid the differential diagnosis in these patients.

## Abbreviations

ICU = intensive care unit; 1-OHMG = 1-hydroxymidazolam glucuronide.

## Competing interests

The author(s) declare that they have no competing interests.

## Authors' contributions

All authors participated in the study design, interpretation of results and manuscript preparation. CMK also performed data collection and analyses.

## Figures and Tables

**Table 1:** Patient characteristics and outcome

Characteristics	Midazolam-positive group (*n *= 13)	Midazolam-negative group (*n *= 13)	Statistics
Admission diagnosis			
Cardiothoracic surgery	8	6	
Severe sepsis	0	4	
Cardiorespiratory arrest	2	1	
General surgery	2	0	
Pancreatitis	1	0	
Acute asthma	0	1	
Perforated duodenal ulcer	0	1	
APACHE II score (day 1; mean ± standard deviation)	19.1 ± 6.6	19.4 ± 7.1	
Renal Impairment (serum creatinine >130 μmol/l or receiving renal replacement therapy)	5	4	
Neurological function intact	10	3	*P *< 0.002^a^
Neurological damage	3	10	*P *< 0.002^a^
All-cause mortality	4 (31%)	8 (62%)	
Median midazolam dose mg/hour (range; 24 hours before cessation)	4 (2–20)	3.5 (2–15)	NS^b^
Median time (range) from midazolam cessation (hrs)	66 (36–120)	68 (36–146)	NS^b^

^a^χ^2 ^test. ^b^Mann–Whitney U-test. APACHE, Acute Physiology and Chronic Health Evaluation.
